# Using Social Network Methods to Test for Assortment of Prosociality among Korean High School Students

**DOI:** 10.1371/journal.pone.0125333

**Published:** 2015-04-27

**Authors:** Jun-Hong Kim, Darryl J. Holman, Steven M. Goodreau

**Affiliations:** 1 Institute of Cross-Cultural Studies, Seoul National University, Seoul, South Korea; 2 Department of Anthropology, University of Washington, Seattle, WA, United States of America; 3 Center for Statistics and the Social Sciences, University of Washington, Seattle, WA, United States of America; 4 Center for Studies in Demography and Ecology, University of Washington, Seattle, WA, United States of America; Peking University, CHINA

## Abstract

Assortative interaction among altruistic individuals is a necessary condition for the evolution of cooperation. The requirement for assortment holds regardless of whether a meta-population is subdivided into distinct and isolated subgroups or has ephemeral boundaries with a high migration rate. The assumption, however, is rarely tested directly. In this paper, we develop a method to test for assortment of prosociality in network-structured data. The method is applied to a friendship network collected from 238 Korean students attending the same high school. A mixing matrix was used to explore the presence of assortative friendship among more prosocial individuals. An exponential random graph model of network structure that accounts for additional observed relational propensities (higher-than-expected number of people nominating no friends) and sampling constraints (upper bound on friendship nominations) found that individual prosociality predicted friendship propensity, and that individuals with higher prosocial scores had a higher probability of befriending other more prosocial individuals. The results reveal that a considerable level of assortment of prosociality characterizes this population.

## Introduction

In evolutionary science, altruism is defined as a behavior that conveys a fitness benefit to the recipient at some cost to the actor [1, 2]. Altruism cannot ordinarily evolve through individual selection because only altruists bear fitness costs, while selfish individuals accrue fitness benefits without the costs. In order for altruism to evolve, additional constraints must be in place, such as assortative interaction among altruists [3, 4], cultural conformity [5, 6] and punishment of norm avoiders [6, 7].

Unless altruistic behavior is inexpensive (i.e. weak altruism), assortment is required for the evolution and maintenance of altruism [3, 4, 8, 9, 10]. Any successful models of cooperation have an assortment feature, whether the model is kin selection, reciprocal altruism or cultural group selection [7]. In kin selection theory, assortment occurs through nepotism toward close relatives. Reciprocal altruism requires exclusion of defectors and preferential interaction with faithful reciprocators. Cultural group selection theory requires conformity to cultural norms and exclusion of individuals that do not conform. The net result is that differences in the frequency of altruists arise among groups. In any of these models, cooperation evolves when the benefits of altruistic behavior preferentially flow toward the actor or other altruistic individuals without exploitation by the self-interested.

The amount of assortative interaction that is required for the evolution of altruism depends on the benefit-cost ratio of altruistic behavior. When other-regarding behavior is not costly to actors, cooperation can be maintained under random interaction (e.g. weak altruism). However, when the cost of altruism is greater (i.e. the fitness benefit to the recipient exceeds the cost to the actor, b>>c), some degree of positive assortment must exist for altruism to proliferate [7]. For example, if virtually every individual plays the defector strategy in an iterated prisoner’s dilemma game, the cooperative strategy can invade this population only through clustering or kinship [11]. Therefore, the success of the altruistic strategy depends upon population structure—that is, how the social interactions are structured. The requirement for assortment holds regardless of whether a meta-population is subdivided into distinct and isolated subgroups or has ephemeral boundaries with a high migration rate [12]. Weak altruism (i.e. a net positive payoff to an actor for that actor’s behavior) cannot evolve under negative assortment conditions [4]. We note that there is no requirement that altruists share an “altruist gene.” Rather, the shared trait can be a cultural phenotype [6].

There are numerous methods to quantify the degree of assortative interaction among actors. All of these have at their core the measure of some trait on individuals, and a determination that those with similar traits share relations more often than expected by chance. In the study of altruism, however, previous work has typically relied on indirect evidence for assortment among altruists. For example, previous work has demonstrated assortment by the presence of a structured population where a group structure is assumed to entail assortment (e.g. [13]) or partner choice (e.g. [14]). In this paper, we adopt a new, flexible method, based on social network analysis, to quantify the nature and levels of assortment from network-structured observations. We then apply the method to an analysis of prosocial assortment within friendship networks in a South Korean high school. We employ a survey instrument that is similar to the “Developmental Assets Profile (DAP)” used by Wilson et al. [15] to measure individual prosociality, and collect additional data on friendship networks and individual demographics.

## Materials and Methods

### Ethics Statement

This study was reviewed and approved by the Human Subject Division of the University of Washington. The UW HSD approved the assent procedure for the adolescent subjects, and waived the need for parental permission (#38874). For each survey participant, assent information was explained in person, and then informed assent was obtained online.

### Subjects

The field research was conducted in Pohang city, South Korea. Pohang is well known for its steel industry and shipbuilding. Social processes in Korea display some key differences from those in Western settings where a disproportionate number of empirical studies of social processes in the realm of behavioral evolution have taken place. First, South Korea is racially and ethnically highly homogeneous. In addition, competition for prestigious jobs and higher education is very intense, which may have a detrimental effect on adolescents’ cooperation and concern for others. For example, a 2009 International Civic and Citizenship Education Survey report ranks South Korea lowest (among 38 countries) on a scale of student’s trust in civic institutions (such as national government, political parties, schools and people in general) and participation in civic activities (such as human rights and environmental organizations) [16].

We conducted a web-based survey of 1st and 2nd grade high school students (these correspond to the 10th and 11th grades in the US) in one high school in Pohang in 2010. The response rate was 83% (462 out of 556). High school students were used as the target population because survey response rates among high school students tend to be higher than that of adults [17], and a high response rate is required for this type of social network analysis [18]. Among 462 participants, 55 were excluded for a failure to answer more than 5 questions, for identical scores on all items (e.g. 1-1-1-1) or for not providing demographic information. The social network analyses were conducted for a single grade (N = 238).

The survey is a modified version of the survey used in Wilson et al.’s [15] study from the Binghamton Neighborhood Project. We included additional demographic and friendship network questions (Table 1). Eight individual prosociality questions measured a respondent’s willingness to maintain a cooperative relationship with other people (e.g. “I am helping to make my community a better place.”). Answers were given on a five-point Likert scale (1 = not at all to 5 = always). In addition to individual prosociality questions, participants were asked to list their seven closest friends with unique identifiers that included names, addresses and home rooms.

**Table 1:** Survey Question.

Subject	Items or Question
Annual Income of your family (if both parents earns money, sum both incomes)	1. Less than $25,000 2. More than $25,000 but less than $40,000 3. More than $40,000 but less than $60,000 4. More than $60,000 but less than $100,000 5. More than $100,000 6. Unable to answer
Father’s education level	1. Elementary school dropout 2. Elementary school graduate 3. Middle school dropout 4. Middle school graduate 5. High school dropout 6. High school graduate 7. College dropout 8. College graduate 9. Postgraduate
Prosociality	1. “I think it is important to help other people.” 2. “I resolve conflicts without anyone getting hurt.” 3. “I tell the truth even when it is not easy.” 4. “I am helping to make my community a better place.” 5. “I am trying to help solve social problems.” 6. “I am developing respect for other people.” 7. “I am sensitive to the needs and feelings of others.” 8. “I am serving others in my community”
Social Network	For your seven closest friends, provide a full name, grade, class and the neighborhood in which he or she lives.

### Statistical methods

For each subject, five types of variables were collected: sex, household income, father’s education level, individual prosociality, and a list of friends. Individual prosociality scores were calculated by taking the average score for 8 prosociality questions (Table 1). Friendship networks were constructed from the friendship nominations. All network ties (or “edges,” in network parlance) were treated as directed (with A’s potential nomination of B considered as a separate random variable from B’s potential nomination of A). Individual (or “node”) attributes thus include four metrics: measures of prosociality, father’s education level, household income and sex.

Our expectation is that positive assortment will occur at higher rates among more prosocial individuals, so that prosocial individuals will have more interactions with other prosocial individuals than with selfish individuals. More precisely, the intensity of assortative interaction can be measured as the difference between the probability of altruists interacting with other altruists and the probability of non-altruists interacting with altruists [7].

Tests of assortment in social network data can be conducted by comparing the frequency of altruist-altruist pairs and non-altruist-altruist pairs. If we assume only two types of individuals are present in a group (more prosocial and less prosocial individuals; see Table 2), then when assortment occurs, ties will be more frequent between pairs of more prosocial individuals (i.e. the type 1 ties) than pairs of more prosocial and less prosocial individuals (i.e. types 2 and 3). The frequency of ties between pairs of less prosocial individuals (i.e. types 4) is not important. When assortment occurs, non-altruists may interact with other non-altruists or they may be excluded from social interaction. We can also extend this to a continuous prosociality variable. For example, when prosocial assortment occurs, we expect pairs of actors to have a higher probability of a tie at higher levels of prosociality.

**Table 2:** Types of ties when two types of individuals are present.

Edges from rows to column	More prosocial individuals	Less prosocial individuals
More prosocial individuals	Type 1 tie	Type 2 tie
Less prosocial individuals	Type 3 tie	Type 4 tie

Edges are directed and from rows to column. For example, tie type 2 includes the ties from more prosocial individuals to less prosocial ones. When our hypothesized form of assortment occurs, the number of tie type 1 should be more frequent than other types of ties.

Our hypothesis does not depend on whether the ties themselves are caused by social selection (prosocial individuals choosing other prosocial individuals), or social influence (prosocial individuals inducing their friends to become more prosocial). Indeed, we cannot distinguish between these given the cross-sectional nature of our data. Our hypothesis also does not depend on the proximate mechanisms of assortment. For example, we are more or less agnostic about whether assortment occurs through nepotism, reciprocity or conformity. Note that we use the term “homophily” in this paper to refer to all positive assortment, regardless of its origin, as is common in the network analysis literature. We refer to its opposite, a prevalence of ties across groups relative to chance, as heterophily.

We analyze the social network data in two ways. For the first analysis, a mixing matrix was used to assess assortative friendship. A 2×2 mixing matrix gives the density of ties between nodes with paired characteristics, in this case two different levels of prosociality [18] (essentially, it is Table 2 with numbers for each of the four tie types filled in). The mixing matrix provides a simple display of the frequencies of friendship types. We converted the individual prosociality variable to a new dichotomous variable, representing more and less prosocial individuals, divided at the median value of individual prosociality (= 3). The group sizes were similar (115 vs. 123) but not identical given the presence of tied prosociality scores. Lastly, we used a chi-square test for assortment. Expected numbers of ties were calculated by the product of three numbers: 1) proportion in the subgroup to which the friendship nominator (a.k.a. “ego”) belongs 2) proportion in the subgroup to which the friendship nomination recipient (a.k.a. “alter”) belongs; and 3) total number of friendships. For example, since less prosocial individuals comprise 51.7% of the population, the expected number of friendships named from one less prosocial individual to another less prosocial individual is 222.5 (= 0.517×0.517×833).

The dichotomous chi-squared tests provide a quick and approximate overview of homophily in social relationships using a statistical method that is widely familiar within the social and biological sciences. However, it has two limitations. One, it requires an arbitrary cutoff, and does not provide robust statistics for (nearly) continuous variables such as the prosociality score in our study. Additionally, the mixing matrix does not properly account for the non-independence resulting from many individuals having multiple friendships. To overcome these inherent problems with the mixing matrix method, we use exponential random graph models (ERGMs [19–23]) to identify variables that predict ties between individuals in the friendship network. ERGMs are a preferred alternative to an analysis of the mixing matrix because the model can control for multiple predictors while also controlling for the non-independence inherent in network structures, and yields similar interpretations. Potential terms in an ERGM include functions of node and tie covariates (such as counts of friendships by prosociality scores of the actors in them) or structural elements, such as counts of triangles or other forms of clustering. (For a list of ERGM terms, see: http://svitsrv25.epfl.ch/R-doc/library/ergm/html/ergm-terms.html.)

Both ERGMs were checked for convergence and for standard Markov chain Monte Carlo diagnostics (confirming that networks simulated from the model fits accurately captured the sufficient statistics to the model), to ensure that the coefficients could be interpreted. Both were also checked for goodness of fit between simulated and observed networks on additional higher-order network statistics to ensure that the posited local network processes were reasonably consistent with the overall emergent observed network. Additional details are given in the Appendix (See S1 and S2 Figs).

## Results

Descriptive statistics for prosociality are given in Table 3. The mean prosociality score (3.09) lies very close to the center of the possible distribution (1.0–5.0), and the individual scores reported cover almost the entire range. The mean prosociality score and its standard deviation are greater for male participants than those for female participants, although not significantly so (2-sided *t*-test, *p* = 0.336).

**Table 3:** Mean scores, standard deviation and ranges on prosociality.

Prosociality	Mean	S.D.	Range
Total population (N = 238)	3.093	0.603	(1.5,5)
Male (N = 101)	3.14	0.666	(1.5,5)
Female (N = 137)	3.059	0.552	(1.75,4.75)

**Fig 1:**
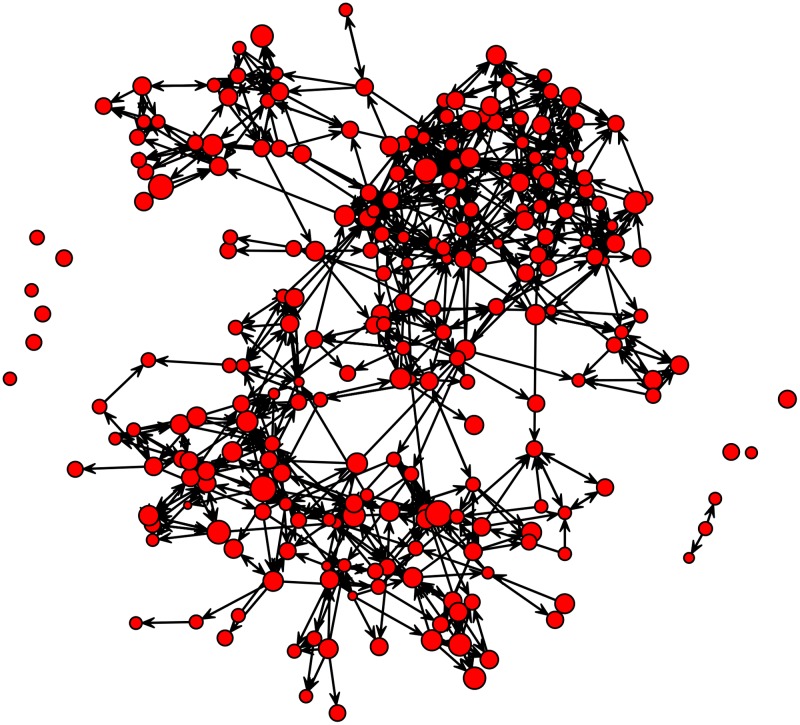
Directed friendship network in a South Korean high school. Circles and arrows show individuals and friendship, respectively. The diameter of the circle shows relative level of individual prosociality (online version in color).

The friendship network (Fig 1) contains 238 individuals and 833 directed ties, implying that students nominated an average of 3.5 friends, and also were nominated as a friend an average of 3.5 times. There were 9 isolates–individuals that named no friends and were not listed by other participants as a friend.

The mixing matrix for our dichotomized prosociality measure is given in Table 4. If assortative interaction is absent between individuals (i.e. a random friendship without regard to prosociality), the four cells in the mixing matrix should have similar counts to the expected (i.e. the expected counts according to the proportion of each subgroup). However, friendship between pairs of more prosocial individuals and friendship from more prosocial individuals towards less prosocial individuals are more frequent than the expected counts. The chi-square goodness-of-fit test (χ^2^ = 6.865, 1 d.f., *p*-value < 0.01) suggests that assortative mixing occurs and can be detected in the dichotomized prosocial variable.

**Table 4:** Mixing matrix between different levels of prosociality.

Edges from rows to column	More prosocial individuals (N = 115)	Less prosocial individuals (N = 123)
More prosocial individuals(N = 115)	216 (194.5)	222 (208)
Less prosocial individuals(N = 123)	199 (208)	196 (222.5)

Numbers outside and within parentheses are observed and expected numbers of friendship, respectively. Expected numbers of edges are the product of three numbers: 1) proportion of subgroup where the friendship is named from, 2) proportion of subgroup where friendship is directed to, and 3) total number of friendship (833). For example, the expected number of friendship among more prosocial individuals is 194.5 (= 0.483×0.483×833).

Two kinds of ERGMs were used to consider assortment by prosociality using the full information in the continuous data. In the process, we could also compare assortment by prosociality to assortment by other node attributes (father’s education and household income), all in the presence of additional structural effects exhibited by the data (for all ERGM terms used in this study, see Table 5; for ERGM codes, see S1 Code.). Common terms in both models (explained below) include counts of ties, sex-homophilous ties, nodes with zero out-ties and triad effects. Since we collected a maximum of seven friendship nominations, the models also contain a constraint such that during model estimation, the space of possible networks considered only includes those in which all nodes have seven or fewer out-ties. This method, of course, cannot recreate the missing information for anyone who would have chosen to nominate more than seven friends in the absence of the restriction; however, it does constrain the model sample space to measure the true effects within the observed data given those constraints.

**Table 5:** ERGM terms, with descriptive names and brief definitions.

ERGM term	Descriptive name	Definition
*edges*	Count of edges	When all other coefficients in the model are zero, this term indicates the log-odds of a tie within a dyad (i.e. between two actors)
*nodematch*	Homophily (discrete)	Uniform homophily for a discrete node attribute. When two nodes have the same value for the attribute, the log-odds of a tie increases (when the *nodematch* coefficient is positive)
*odegree(0)*	Nodes with zero out-tie	A propensity to be a “loner”—i.e. to make no friendship nominations
*absdiff*	Homophily (continuous)	Homophily (or heterophily) for a continuous node attribute. The greater the difference in two nodes’ values for an attribute, the greater their log-odds of forming a tie (when *absdiff* coefficient is positive). Typically the coefficient is negative, indicating that greater similarity corresponds to greater tie probabilities.
*nodeocov*	Out-popularity	Effect of a node attribute on the log-odds of outgoing ties
*nodeicov*	In-popularity	Effect of a node attribute on the log-odds of incoming ties
*edgecov*	Node attribute product effects	*edgecov* allows for a numerical attribute to be assigned to each edge. In our usage, that value equals the product of the two adjacent node’s attributes; the higher that product, the higher the log-odds of a tie (when the *edgecov* coefficient is positive).
*gwesp*	Triad effect	This models the additional propensity for two nodes to form a tie, for each relational partner that they have in common (i.e. for each triangle that will be created when they form a tie). This effect is rarely linear in practice, but tends to exhibit diminishing marginal returns—each additional shared partner adds a smaller amount to the log-odds. The alpha parameter controls the rate of that decline, according to a geometric function, yielding the name “geometrically weighted edgewise shared partners,” or *gwesp*.

The interpretation of the coefficient on the count of ties is similar to an intercept in multivariate analysis. When all other coefficients in the model are zero, this term indicates the log-odds of any tie. The *sex-homophily* term produces a coefficient that, when exponentiated, is the conditional odds ratio of a tie between dyads if the two actors are of the same sex. If dyads are of different sexes, then there is no increase. A term for nodes with zero out-ties was included to estimate a specific propensity to be a “loner”—that is, to nominate no friends—since these were found to be highly over-represented in the data relative to any reasonable null model (51 out of 238 nodes). This is a fairly typical pattern for adolescent friendship networks, and may include both true “loners” and those who simply didn't bother to fill out the list of friends in the survey or who nominated others who did not participate in the survey [24]. The triad effect term was included to account for transitivity, which is also a common attribute of adolescent friendship networks. This occurs when a friend of a friend in turn becomes a friend, perhaps as a result of increased opportunities for social interaction. Triad effects can interact with homophily in subtle ways [24]; inclusion of both terms in the model together allows us to tease apart their effects. Without including triad effects, the homophily parameters would also incorporate effects of triad formation among individuals. In essence, we are separately controlling for a propensity of an individual to befriend a friend’s friend, and assortment by prosociality.

### ERGM 1

To motivate the additional terms in this model, we refer to the classification of ties presented in Table 2. Previously, we defined assortment in terms of the relative preponderance of type 1 ties compared to type 2 and 3 ties. More numerous ties from and to prosocial individuals could be attributed to at least two phenomena: popularity and homophily. Here, popularity means that prosocial individuals tend to have more incoming and/or outgoing ties than others do, regardless of whom those ties are with. Homophily means that people with similar level of prosociality tend to associate with each other. Table 6 shows the nature of coefficients in a hypothetical ERGM containing popularity and homophily terms based on a dichotomized prosociality metric. In this case, θ_2_ and θ_3_ reflect main effects for popularity on prosocial individuals (for out-ties and in-ties, respectively); while θ_4_ reflects the additional effect for ties that are homophilous on prosociality. In our model, however, we are able to extend the analysis beyond a simple dichotomization of prosociality and use the full prosociality score. In this case, θ_2_ and θ_3_ reflect increases in log-odds for each unit increase in the prosociality score for the relevant actor. For this model, the θ_4_ parameter is the effect of the absolute difference in the prosociality score of the two nodes, such that a negative coefficient means that nodes that are more similar in score are more likely to have a relationship.

**Table 6:** Parameterization of the exponential random graph model (ERGM).

Edges from rows to column	More prosocial individuals	Less prosocial individuals
More prosocial individuals	θ_1_+ θ_2_ + θ_3_ + θ_4_ *	θ_1_+ θ_2_
Less prosocial individuals	θ_1_ + θ_3_	θ_1_+ θ_4_

* θ_1_ is the default level of friendship without popularity and homophily; θ_2_ is the effect of prosociality on outgoing ties (positive means individuals with higher prosociality have more numerous outgoing edges); θ_3_ is the effect of prosociality on incoming edges; θ_4_ is the effect of homophily (association with others exhibiting a similar level of prosociality).

The result of model 1 (Table 7) indicates that, after controlling for homophily by sex, propensity to be a loner and triad effects, two assortment terms are significant: out-popularity by prosociality and homophily by household income. Although both terms are significant, considering the possible ranges of both variables are similar, the effect of homophily by household income (0.083) is relatively smaller than the effect of out-popularity by prosociality (0.341). We note that the homophily by prosociality term is positive, which is the reverse of our expectation, although it is not significant. If the mixing matrix is a correct description, this means that the high frequency of type 1 ties results from prosocial individuals having more frequent outgoing ties in general.

**Table 7:** ERGM estimates (model 1).

ERGM terms	Estimate	Std. Error	*p*-value
*Edges*	-9.105	1.337	<0.001***
Sex (*nodematch*)	3.826	0.378	<0.001***
Prosociality (*nodeicov*)	-0.015	0.074	0.84
Prosociality (*nodeocov*)	0.341	0.199	0.087*
Prosociality (*absdiff*)	0.149	0.091	0.103
Household Income (*nodeicov*)	0.013	0.023	0.579
Household Income (*nodeocov*)	- 0.036	0.039	0.354
Household Income (*absdiff*)	- 0.083	0.036	0.021**
Father’s education level (*nodeicov*)	- 0.037	0.04	0.349
Father’s education level (*nodeocov*)	- 0.042	0.064	0.511
Father’s education level (*absdiff*)	- 0.038	0.052	0.463
*odegree*(0)	1.485	0.354	<0.001***
Triad effect (*gwesp*)	3.444	0.122	<0.001***

* Significant at p < 0.1;

** Significant at p < 0.05;

*** Significant at p < 0.001.

### ERGM 2

**Fig 2:**
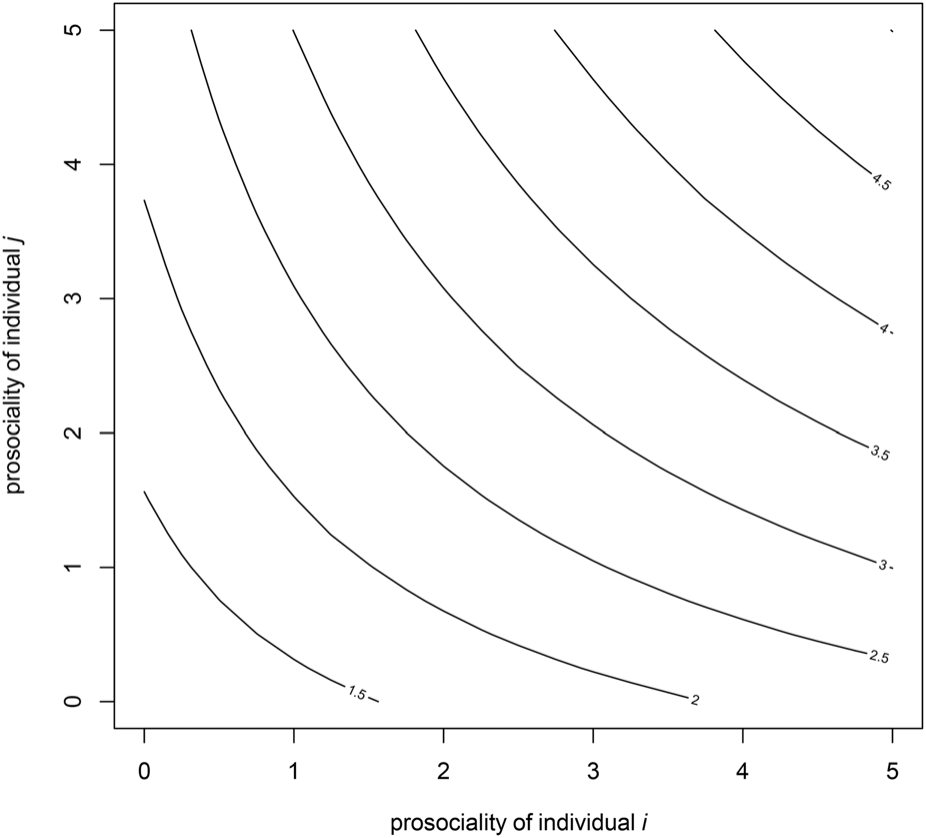
Contour plot for the prosocial edge covariate for ERGM 2. The numbers on the *x* and *y* axes correspond to the prosociality scores of the actors *i* and *j* and the corresponding point in the matrix represents the *edgecov* value for dyad (i, j), which equals the square root of the product of the two actors’ prosociality.

The first ERGM can separate out three independent effects of prosociality on friendship. The disadvantage of the first model is that it does not test whether type 1 ties are uniquely more frequent than type 2 and 3 ties. In order to test assortment with a single value, we define a new measure for each pair of actors, the square root of the product of their two prosociality scores (Fig 2). This measure increases with increasing prosociality for either actor, but the product ensures that it increases most when both actors are highly prosocial. Thus, it emphasizes homophily specifically at the highly prosocial end of the scale. In contrast, the homophily term in model 1 considers homophily in the whole range of prosociality where the positive coefficient may be due to a strong homophily among non-prosocial individuals in addition to, or instead of, homophily among prosocial individuals. We take the square root to scale the metric more like our other measures (i.e. from 1 to 5).

The ERGM 2 (Table 8) results confirm assortment by prosociality. Using this framework that isolates homophily at the more prosocial end from homophily at the less prosocial end of the scale, we indeed see a significant and positive effect. The general interpretation of model 2 is the same as model 1. The only significant and positive term among assortment terms is the prosociality-product homophily term. In addition, a significant but relatively smaller effect of heterophily by father’s education level also was found.

**Table 8:** ERGM estimates (model 2).

ERGM terms	Estimate	Std. Error	*p*-value
*Edges*	-9.137	0.268	<0.001***
Sex (*nodematch*)	3.808	0.199	<0.001***
Prosociality (*edgecov*)	0.211	0.044	<0.001***
Household income (*edgecov*)	-0.002	0.015	0.887
Father’s education (*edgecov*)	-0.045	0.019	0.02**
*odegree*(0)	1.171	0.234	<0.001***
Triad effect (*gwesp*)	3.367	0.065	<0.001***

** Significant at p < 0.05;

*** Significant at p < 0.001.

## Discussion

We present a method for quantifying the extent of assortative interaction in network-structured observations. The method controls for other types of homophilous relationships (e.g. sex, income), separately controls for triad formation and popularity, and specifically tests for a higher probability of assortative ties among more prosocial individuals (e.g. type 1 ties). The method also controls for observed relational propensities like a higher-than-expected number of people nominating no friends, and sampling constraints like a ceiling on the number of ties for an individual. ERGM 1 found that prosocial individuals were more likely to send ties. In addition, ERGM 2 found greater homophily at the prosocial end of scale. Thus, in these Korean highschool students, the type of prosocial assortment exists that would be necessary for the evolution of prosociality.

We applied the method using friendship as an example of a cooperative relationship that frequently arises outside the context of kinship. While friendship is frequently mentioned as a model of reciprocity, human friendship does not strictly conform to direct reciprocity models [25, 26]. With a friendship, one can lose track of past interactions or forget past benevolent behaviors toward a friend. In order to evaluate the structure of cooperation, we would, ideally, consider all possible prosocial and antisocial interactions. At the least, we would need to discern the difference between friendship and the many other kinds of prosocial interactions. However, as a proxy for reciprocal altruism, friendship may be a good starting point to further explore the structure of cooperation.

The self-reported nature of the prosociality data is a potential weakness, as self-reports may include systematic biases that are difficult to evaluate. Ideally, we would use more direct measures of prosociality, perhaps observations of volunteerism, reputation, or charitable giving. Alternatively, survey data could also be supported by ethnographic and behavioral observations. As is, self-reported metrics provide a useful starting point.

We are agnostic about the proximate mechanisms through which assortative friendships occur. Specifically, assortment could take place either through the formation of relationships with other prosocial individuals (social selection) or through the modification of individuals’ behaviors based on those of their friends (social influence). As shown experimentally, a person might change their prosociality through a social learning process such as conformity [27]. On the other hand, there is contrary experimental evidence that dynamic rather than static network formation (i.e. social selection) promotes cooperation [28]. However, using a cross-sectional survey, we have no way to differentiate between these two mechanisms; a longitudinal study would be required. For our purposes, however, the question is peripheral, since altruism can evolve as long as an assortative interaction exists, regardless of the direction of causality.

Our work hypothesized the presence of homophily, and we did indeed detect some forms of this. However, we also detected some significant forms of its opposite, heterophily, in some models. The latter includes household income (in ERGM 1) and father’s education (in ERGM 2). This suggests that friendship may involve some forms of complementarity and not just similarity in individual attributes. For example, division of labor might favor interactions where each participant has different knowledge and skills. The question of which types of complementarity or homophily are important in friendship still need to be confirmed empirically, although there is some evidence that homophily seems to be a pervasive factor in determining friendship [29].

A study among the Hadza using social network methods also found that altruists tend to form ties with other altruists [30]. The study also found that non-altruists tend to form ties with other non-altruists, a finding that is not directly predicted by evolutionary theory, but can arise as the complement of assortment among prosocial individuals. The Hadza study found increases in the odds of a social tie using logit regression in which only dyad attributes can be included as control variables. On the other hand, our alternative method based on ERGMs, can address dependence between ties and can control for larger structural features of a network.

A recent study in two East African villages that used social network methods found that individuals who are trusty or trustworthy (measured by a trust game) tend to have more incoming ties and connect many other pairs, directly or indirectly (i.e. high betweenness centrality) [31]. Ties were connected to individuals from whom one would prefer to ask social or political advice. Centrality measures are useful for assessing alternatives to social evolutionary models, like for models based in social capital theory. High centrality does not imply homphily, and therefore cannot be used alone to test the assumption of assortative interactions among more prosocial individuals that is fundamental to establishing the evolution of altruism.

One last issue regarding dyadic friendship interaction is the distinction between popularity and homophily. Homophily by prosociality is a necessary condition for the evolution of strong altruism, while the popularity of prosocial individuals is not [4]. This is because heterophilous interactions are often avenues for exploitative relationships by selfish individuals. Homophily itself is not enough if homophily predominantly occurs among non-prosocial individuals. Assortative interaction among non-prosocial individuals is not directly connected with the evolution of altruism; only homophily among prosocial individuals is relevant. In this vein, the second ERGM quantified how frequently homophilous ties occurred among prosocial individuals (type 1 tie) relative to other types of social interaction (type 2, 3, and 4 ties).

Lastly, we still do not know whether homophily or popularity characterizes social interaction in real life. We have evidence of both homophily and popularity of prosocial individuals, while the Hadza social network gave evidence for homophily but not popularity [30]. More cross-cultural research should address this question.

## Conclusion

We applied social network analytical methods for testing and quantifying the assortative interaction condition necessary, but not sufficient, for the evolution of strong altruism. Previous analyses have used social network analyses to explore the relationship between centrality measures and altruistic propensities [30, 31]. But centrality measures alone are not sufficient to show an interaction preference among more altruistic or prosocial individuals. The exponential random graph models framework can be used with data on interaction networks to directly quantify the extent of assortment by prosociality.

When applied to a sample of Korean school students, the method revealed significant levels of assortment among highly prosocial individuals specifically. Highly prosocial individuals tended to have more friends, particularly friends who were more prosocial, while household income and father’s education level had relatively small influences on friendship. This finding suggests that the pattern of friendship formation in a Korean high school satisfies the necessary (but not sufficient) condition for the evolution of altruism.

## Supporting Information

S1 Code The R code to implement the ERGMs. (DOCX)

S1 Fig Goodness-of-fit plots for ERGM 1. Goodness-of-fit plots compare network statistics for an observed network to those simulated from a given model. This allows for a visual or statistical comparison of the degree to which that model captures aspects of observed network structure. In these plots, the dark line represents the statistics for the observed network, and the boxplots represent the range of the same statistics over 100 simulated networks for that model. The y-axis plots the proportion of the relevant unit (nodes, edges, or dyads) possessing the value of the statistic that is listed on the x-axis. Minimum geodesic distance is the length of the shortest path between two nodes; “NR” indicates that two nodes are not reachable; i.e, there is no path of any length connecting them. In-degree and out-degree reflect the number of in-ties and out-ties a node has. Edgewise shared partners measures the count of partners that two nodes have in common, for all sets of nodes that are ties. It is equivalent to the number of triangles each edge is in; it is thus a measure of local clustering. While local effects such as in-degree and out-degree are well captured, our models could not match the geodesic distribution and the ESP distribution. However, this limitation is not a crucial one, since these higher-order aspects of network structure are not really our prime focus.
(PDF)

S2 Fig Goodness-of-fit plots for ERGM 2. (PDF)

S1 File Friendship network data. Short description for Rdata objects. **1**. "friendship.net": Friendship network data which contains 238 individuals and 833 directed friendship. For each individual (i.e node), five attributes are present: sex, household income, father’s education level, measures of prosociality, and a list of friends. All network ties are directed (with A’s potential nomination of B considered as a separate random variable from B’s potential nomination of A). **2**. "father.education.product.sqrt": For each dyad, the square root of the product of their two father's education scores. **3**. "household.income.product.sqrt": For each dyad, the square root of the product of their two household income scores. **4**. "prosociality.product.sqrt": For each dyad, the square root of the product of their two prosociality scores. **5**. "model.01.rev": The result of model 1. **6**. "model.02.rev": The result of model 2.
(ZIP)
