# Supplementary material for: Using Social Network Methods to Test for Assortment of Prosociality among Korean High School Students
Source: PLoS One. 2015 Apr 27;10(4):e0125333. doi: 10.1371/journal.pone.0125333 (PMC4411050; doi:10.1371/journal.pone.0125333)
Supplement: S1 Code — (DOCX) [file pone.0125333.s001.docx]

Code for model 1

model.01 <- ergm(friendship.net ~ edges + nodematch("sex") + nodeicov("individual.prosociality") + nodeocov("individual.prosociality") + absdiff("individual.prosociality") + nodeicov("household.income") + nodeocov("household.income") + absdiff("household.income") + nodeicov("father.education") + nodeocov("father.education ") + absdiff("father.education") + odegree(0) + gwesp(alpha=0.1, fixed= TRUE), constraints=~bd(maxout=7))

Code for model 2

model.02 <- ergm(friendship.net ~ edges + nodematch("sex") + edgecov(prosociality.product.sqrt) + edgecov(household.income.product.sqrt) + edgecov(father.education.product.sqrt) + odegree(0) + gwesp(alpha=0.1, fixed= TRUE), constraints=~bd(maxout=7))
