# Supplementary figures and images for: Using Social Network Methods to Test for Assortment of Prosociality among Korean High School Students
Source: PLoS One. 2015 Apr 27;10(4):e0125333. doi: 10.1371/journal.pone.0125333 (PMC4411050; doi:10.1371/journal.pone.0125333)

proportion of dyads

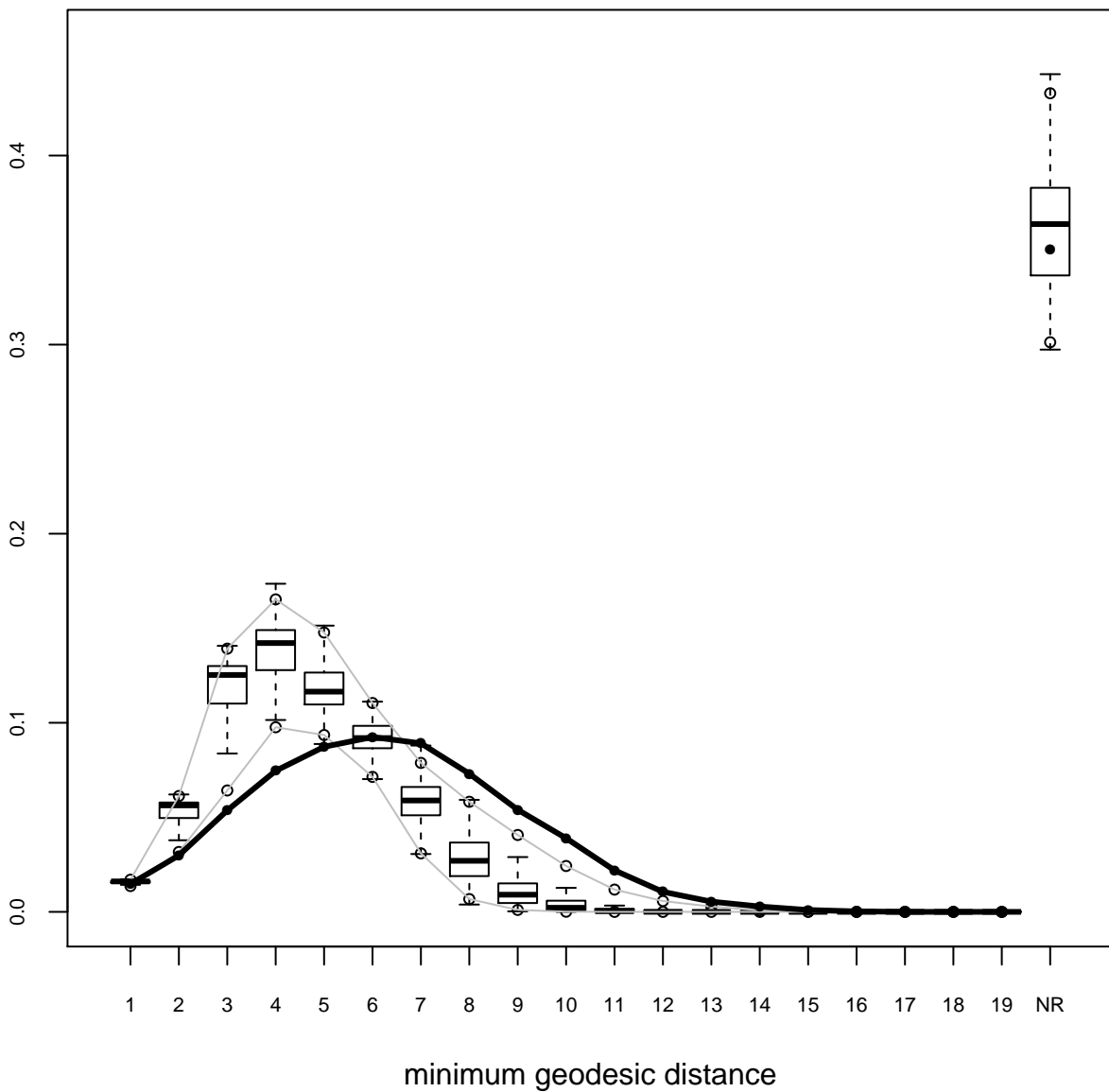

proportion of nodes

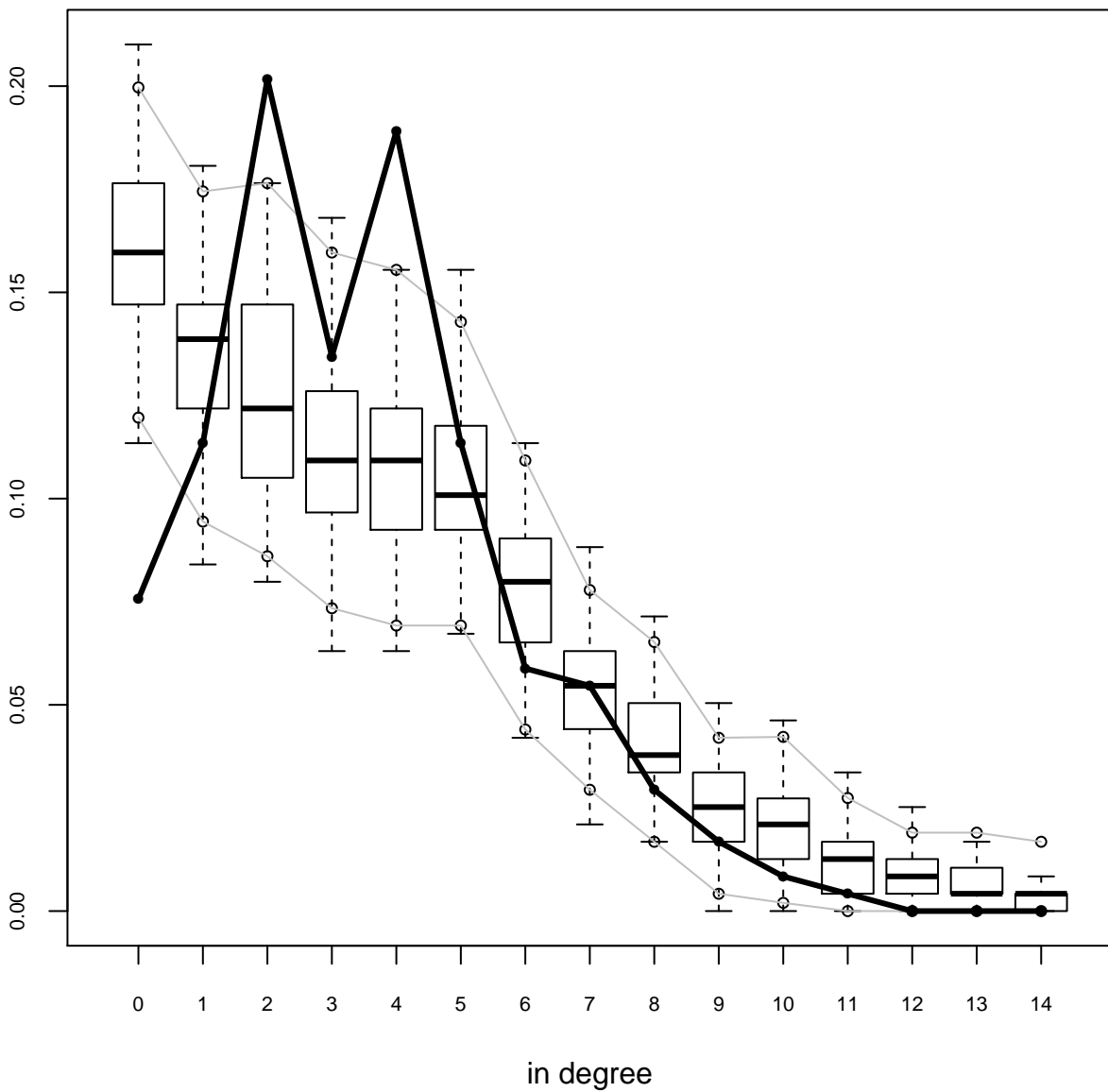

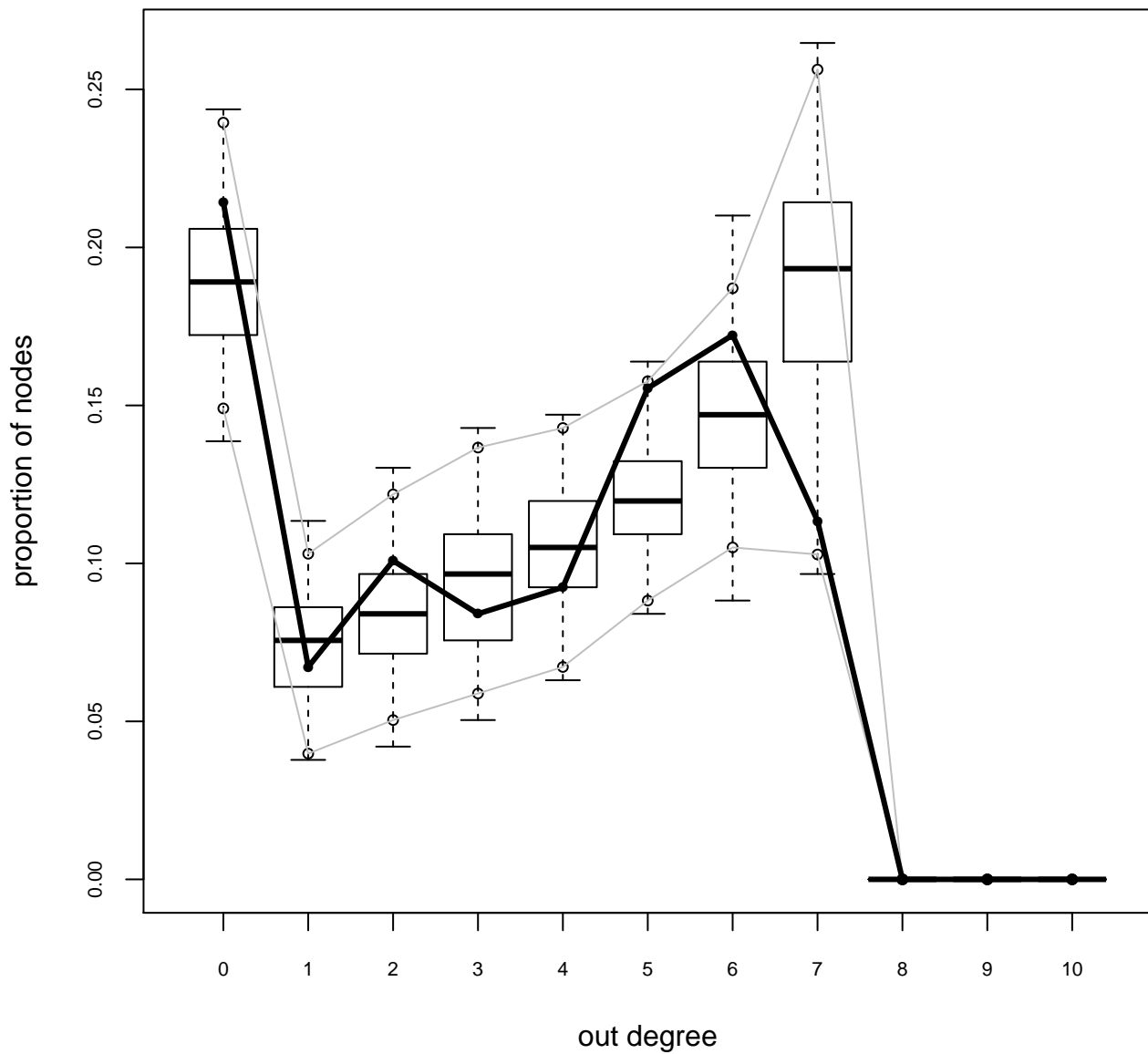

# Goodness-of-fit diagnostics

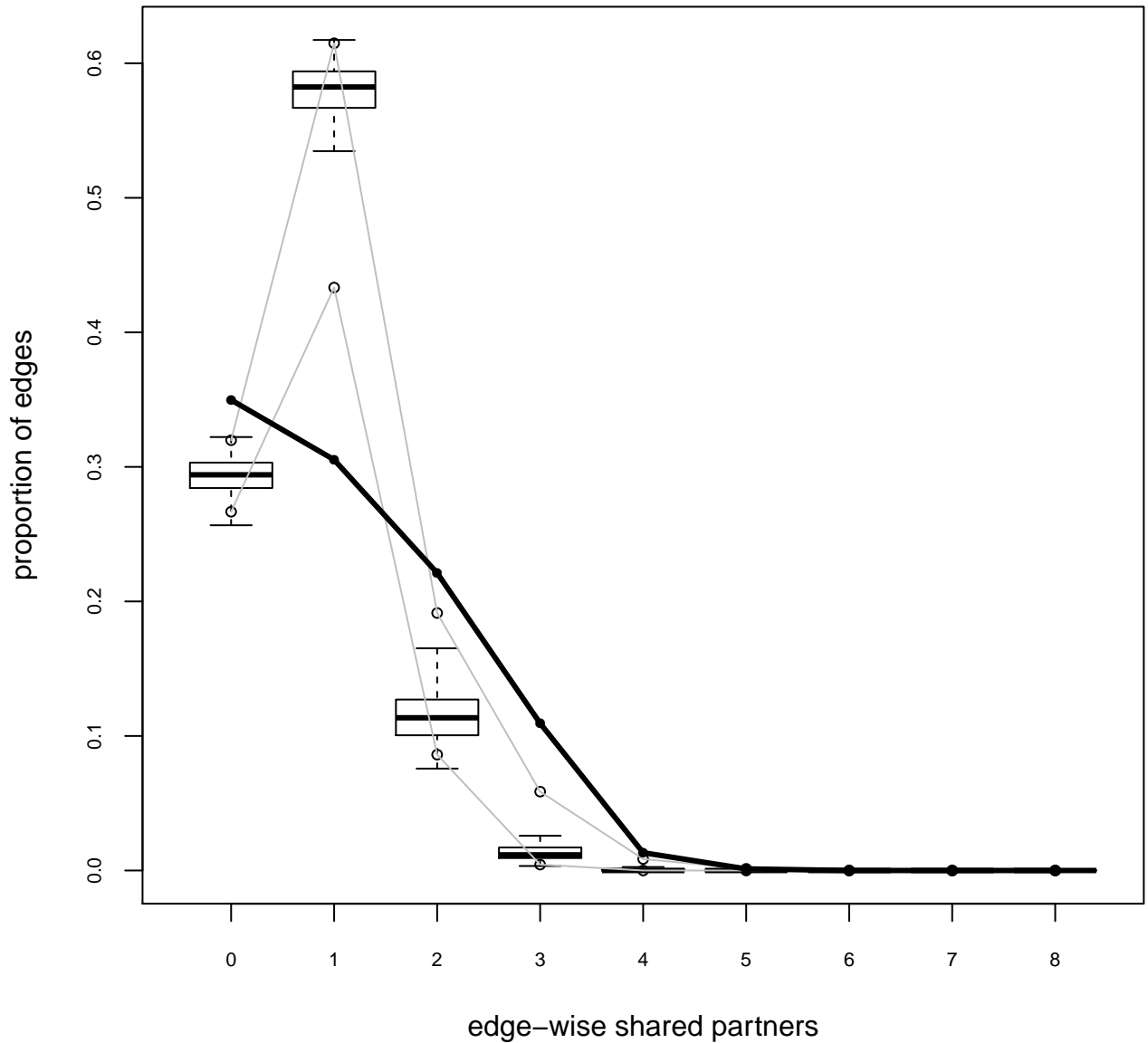

Supplement: S1 Fig — Goodness-of-fit plots compare network statistics for an observed network to those simulated from a given model. This allows for a visual or statistical comparison of the degree to which that model captures aspects of observed network structure. In these plots, the dark line represents the statistics for the observed network, and the boxplots represent the range of the same statistics over 100 simulated networks for that model. The y-axis plots the proportion of the relevant unit (nodes, edges, or dyads) possessing the value of the statistic that is listed on the x-axis. Minimum geodesic distance is the length of the shortest path between two nodes; “NR” indicates that two nodes are not reachable; i.e, there is no path of any length connecting them. In-degree and out-degree reflect the number of in-ties and out-ties a node has. Edgewise shared partners measures the count of partners that two nodes have in common, for all sets of nodes that are ties. It is equivalent to the number of triangles each edge is in; it is thus a measure of local clustering. While local effects such as in-degree and out-degree are well captured, our models could not match the geodesic distribution and the ESP distribution. However, this limitation is not a crucial one, since these higher-order aspects of network structure are not really our prime focus. (PDF) [file pone.0125333.s002.pdf]

proportion of dyads

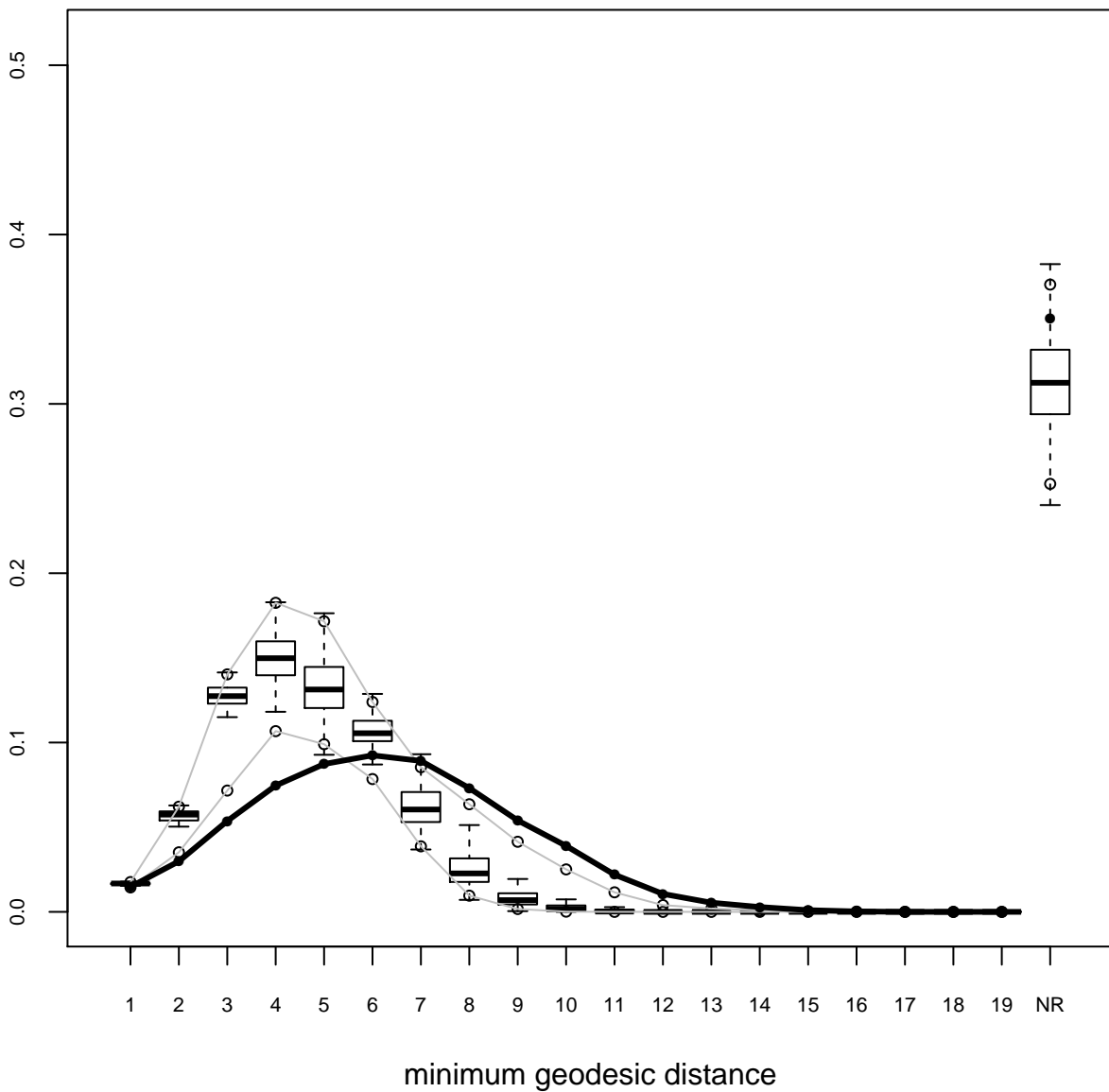

proportion of nodes

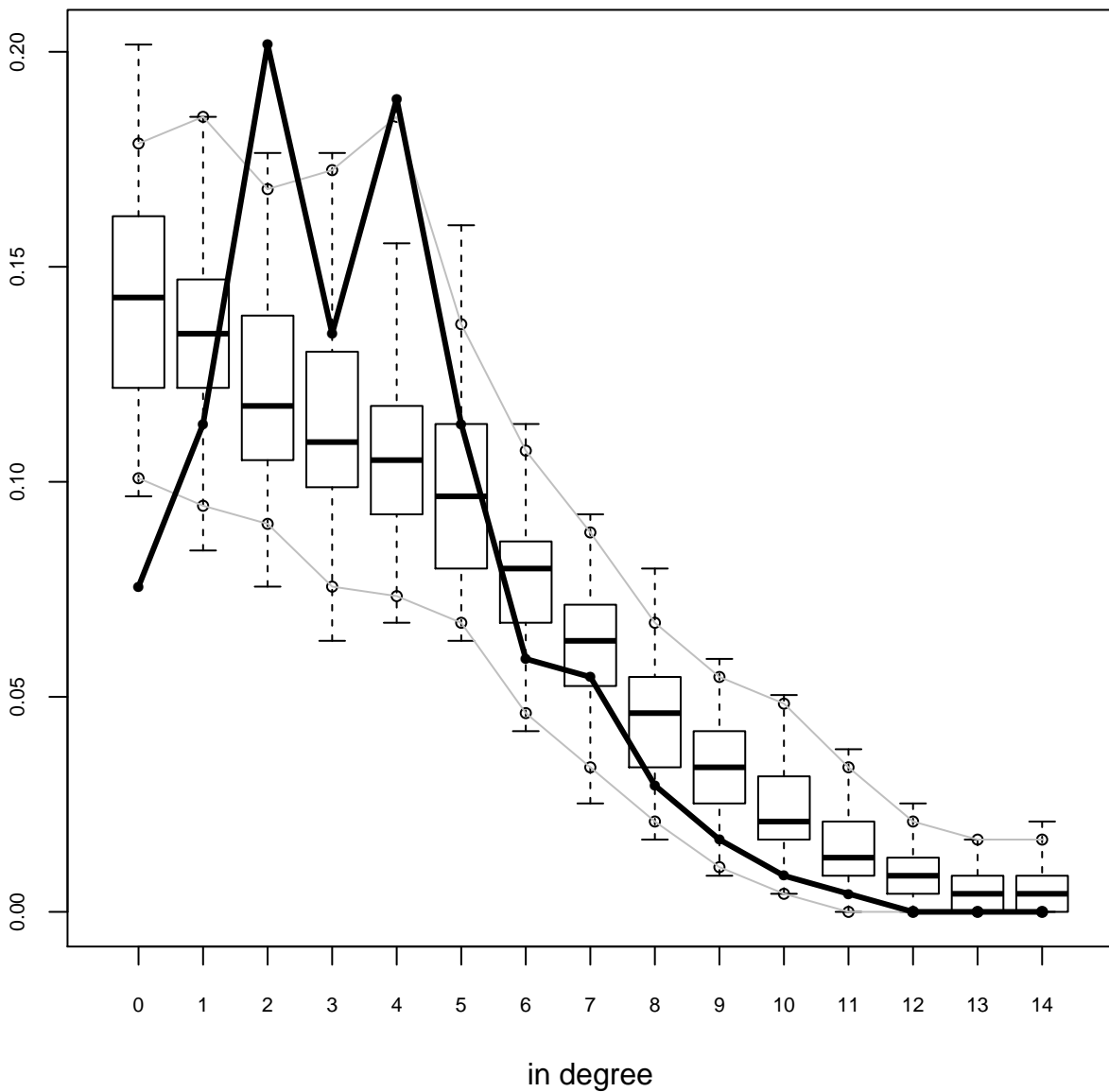

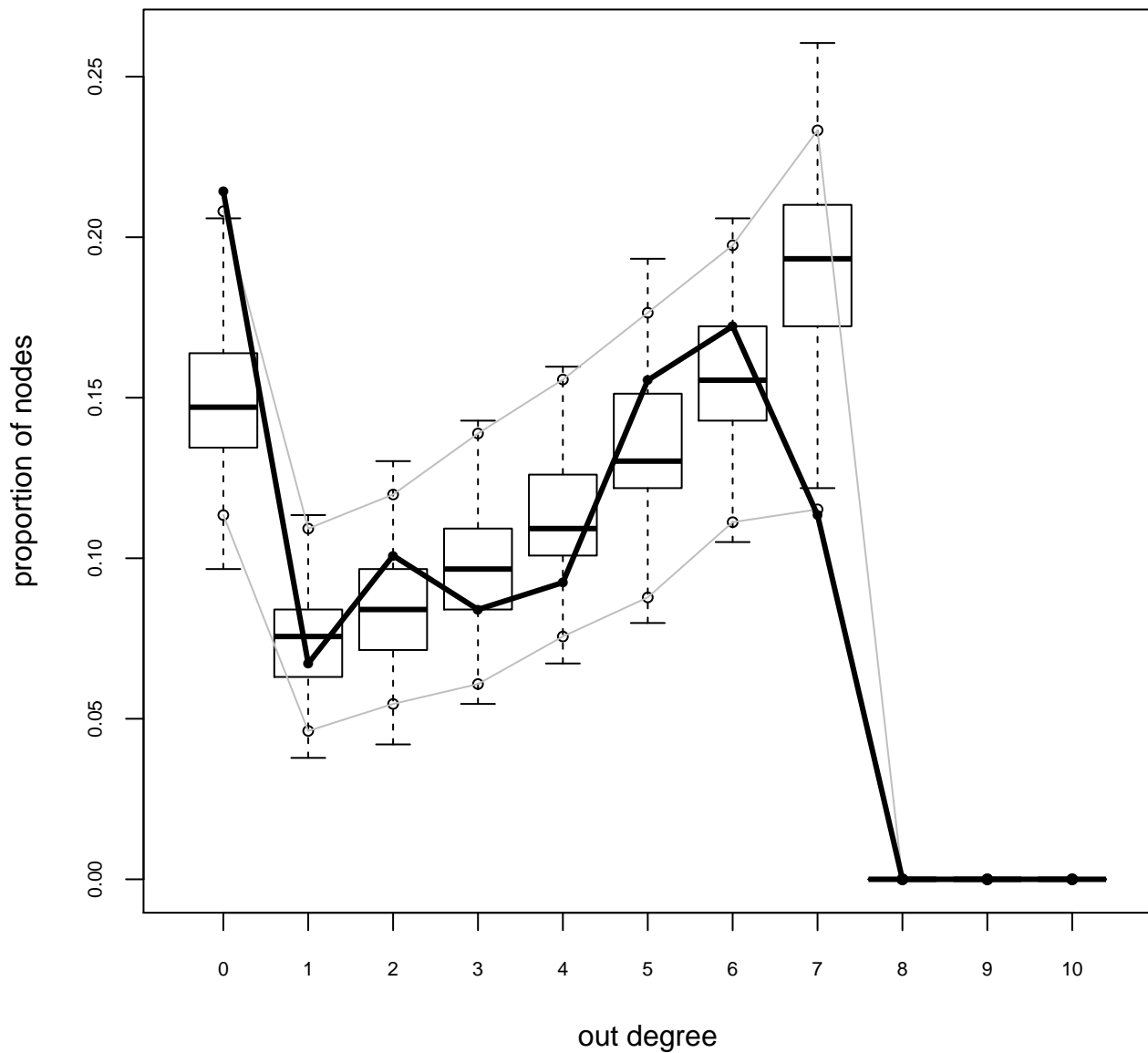

# Goodness-of-fit diagnostics

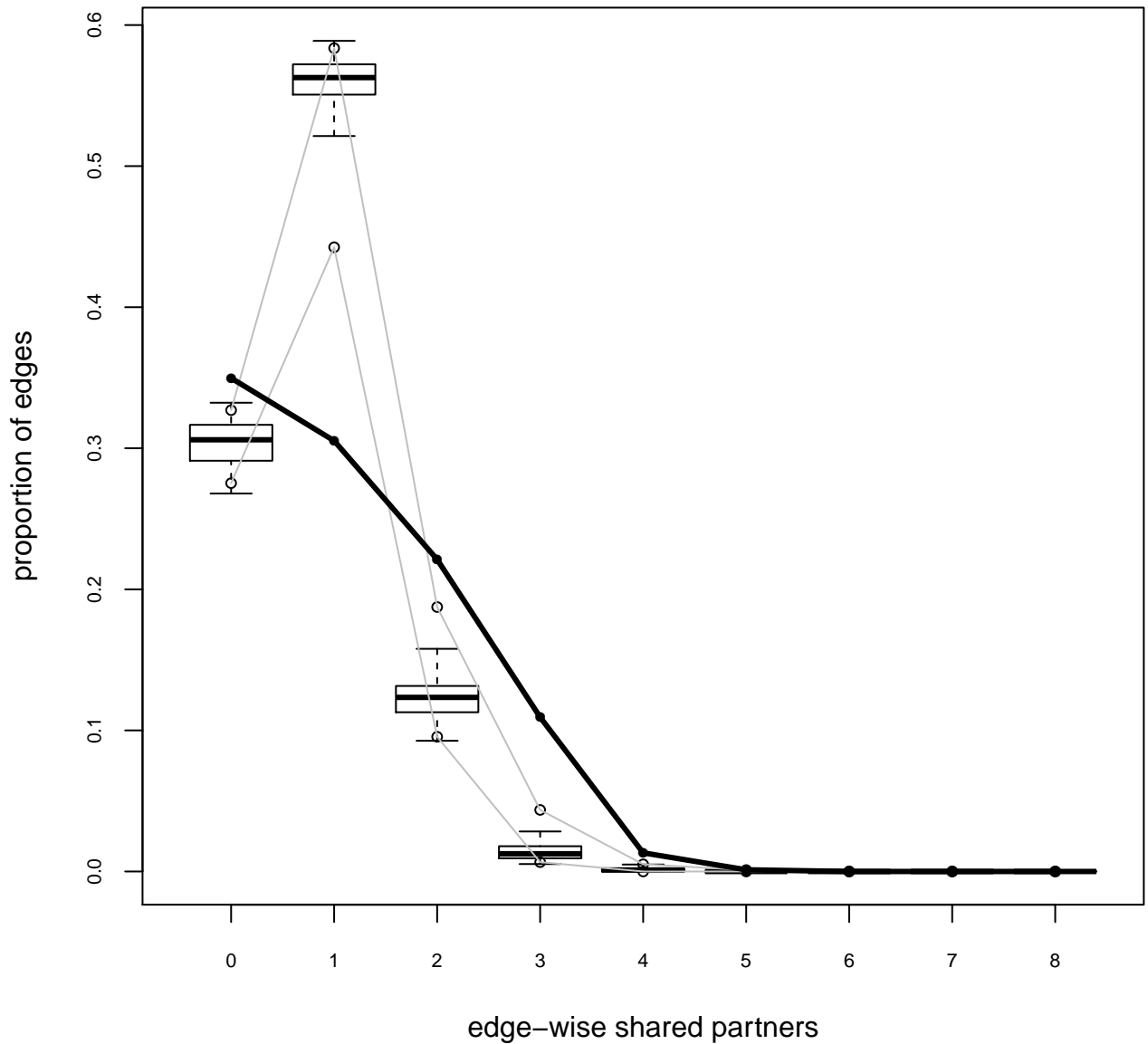

Supplement: S2 Fig — (PDF) [file pone.0125333.s003.pdf]
